# Development of a microsphere-based immunoassay for the serological diagnosis of equine trypanosomosis

**DOI:** 10.1038/s41598-022-05356-y

**Published:** 2022-01-25

**Authors:** Mylène Verney, Morgane Gautron, Charlène Lemans, Alain Rincé, Aymeric Hans, Laurent Hébert

**Affiliations:** 1 Unité PhEED, Laboratoire de Santé Animale, Site de Normandie, ANSES, RD675, 14430 Goustranville, France; 2 Unité de Recherche Risques Microbiens U2RM, Normandie-Université, UNICAEN, Caen, France

**Keywords:** Immunological techniques, Infectious-disease diagnostics, Parasitology

## Abstract

*Trypanozoon* infections in equids are caused by three parasite species in the *Trypanozoon* subgenus: *Trypanosoma equiperdum*, *T. brucei* and *T. evansi.* They are respectively responsible for infectious diseases dourine, nagana and surra. Due to the threat that *Trypanozoon* infection represents for international horse trading, accurate diagnostic tests are crucial. Current tests suffer from poor sensitivity and specificity, due in the first case to the transient presence of parasites in the blood and in the second, to antigenic cross-reactivity among *Trypanozoon* subspecies. This study was designed to develop a microsphere‐based immunoassay for diagnosing equine trypanosomosis. We tested beads coated with eight *Trypanosoma* spp. recombinant antigens: enolase, GM6, PFR1, PFR2, ISG65, VSGat, RoTat1.2 and JN2118HU. Of these, GM6 was identified as the best candidate for the serological diagnosis of *Trypanozoon* infections in equids. Using a receiver operating characteristic (ROC) analysis on 349 equine sera, anti-GM6 antibodies were detected with an AUC value of 0.994 offering a sensitivity of 97.9% and a specificity of 96.0%. Our findings show that the GM6 antigen is a good target for diagnosing equine trypanosomosis using a microsphere‐based immunoassay. This promising assay could be a useful alternative to the official diagnostic tool for equine trypanosomosis.

## Introduction

More than 117 million equids contribute worldwide to the livelihood of many households in the vast community of equine owners^1^. Equids have important socioeconomic roles either for their agricultural production, transport and traction activities, or for equestrian businesses with international exchanges including sales, reproduction and sporting events^2^. In this context, it is necessary to prevent the spread of equine infectious diseases to maintain horse trading worldwide^3–5^. Equine trypanosomoses correspond to a set of pathologies caused by a protozoan of the *Trypanosoma* genus. They share a similar course of infection, with non-pathognomonic clinical signs^6^. Their main causative agents are parasites of the *Trypanozoon* subgenus, including *Trypanosoma* (*T.*) *brucei* for nagana, *T. equiperdum* for dourine and *T. evansi* for surra^6^. As the transmission of *T. brucei* requires the presence of tsetse flies (*Glossina* spp.), nagana is confined to sub-Saharan Africa^7^. In contrast, the sexual transmission of *T. equiperdum* during coïtus and the mechanical transmission of *T. evansi* by haematophagous flies have allowed the dissemination of dourine and surra worldwide^8,9^. Recent outbreaks in non-endemic countries show the major significance to the international horse trade of preventing the spread of these diseases^10,11^. Owing to their closely-related genetic and polyphyletic origins, at least four clades of monomorphic *Trypanozoon* have been identified: *T. equiperdum* type OVI, *T. equiperdum* type BoTat, *T. evansi* type A, and *T. evansi* type B^12,13^. Several diagnostic tools are recommended by the World Organization for Animal Health (OIE) to detect infection by *Trypanozoon*, however serological methods generally suffer from a lack of specificity due to the use of crude antigens from trypanosomes, which may lead to cross-reactivity^14,15^.

Serological assays based on microspheres have raised much interest among the scientific community since the twenty-first century^16,17^, notably the xMAP® technology developed by Luminex® (Luminex Corp., Austin, TX), which combines the benefits of flow cytometry and enzyme-linked immunosorbent assay (ELISA) using smaller sample volumes and faster, automated tests. Another advantage of xMAP® is its multiplexing capability, offering simultaneous detection of up to 50, 100 or 200 analytes for MAGPIX, Bio-Plex200 or FLEXMAP 3D devices respectively. Technically, indirect immunoassays using xMAP® technology may be used to detect antibodies through capture antigens coated on microspheres similar to a microsphere-based ELISA. The potential of this strategy has been demonstrated in particular by Nzou, et al.^18^ who developed an immunoassay for human African trypanosomiasis screening.

In this study, we tested the relevance of eight antigens that were either specific to one clade or common to *Trypanozoon* for developing a microsphere-based method of serological detection for equine infections by *Trypanozoon*. Our results showed that detecting anti-GM6 antibodies using xMAP® technology is a suitably sensitive and specific diagnostic tool for *Trypanozoon* infections in equids.

## Results

### Selection of eight antigens of interest

Eight antigens were selected due to their immunogenic potential for the diagnosis of *Trypanozoon* infections in equids using xMAP® technology (Table 1). Five out of eight antigens were common to the *Trypanozoon* subspecies: *i)* enolase (XP_822542), found in the secretome of trypanosomes and reactive to nanobodies; *ii)* GM6 (Tbg972.11.1200), a flagellar associated protein; *iii)* PFR1 (XP_844021.1) and *iv)* PFR2 (ACP74157.1), both involved in the paraflagellar rod structure; and *v)* the invariant surface glycoprotein ISG65 (XP_011771746), found in the antigen coat of *Trypanozoon* parasites. In order to potentially differentiate between *Trypanozoon* infections, three variable surface glycoproteins (VSGs) described as specific to one clade were included in the study: the RoTat1.2 VSG (AEL79575.1) specific to *T. evansi* type A, the JN21118HU VSG (AJ870487) specific to *T. evansi* type B and an atypical VSG (VSGat-SCU70408) described as specific to *T. equiperdum* parasites without detailing whether it is specific to *T. equiperdum*  type BoTat or *T. equiperdum* type OVI clades.

**Table 1:** Description of the selected recombinant antigens.

Selected antigen	Species specificity	Protein ID	Function	N-ter fusion	Antigen region	C-ter fusion	Reference
Enolase	***Trypanozoon***	XP_822542	Metabolic protein	-	1–429	LEHHHHHH	^37^
GM6		Tbg972.11.1200	Flagellar associated protein	MASWSHPQFEKGALEVLFQGPGYQDP	1962-2029^a^	VDAAAELALVPRGSSAHHHHHHHHHH	^18^
PFR1		XP_844021.1	Paraflagellar Rod	-	1–589	LEHHHHHH	^38^
PFR2		ACP74157.1	Paraflagellar Rod	-	1–600	LEHHHHHH	^39^
ISG65		XP_011771746	Invariant surface glycoprotein	MASWSHPQFEKGALEVLFQGPGYQDP	20–385	VDAAAELALVPRGSSAHHHHHHHHHH	^18^
VSGat	***T. equiperdum***	SCU70408	Variant surface glycoprotein	M	18–470	LEHHHHHH	^40^
RoTat1.2	***T. evansi* type A**	AEL79575.1	Variant surface glycoprotein	M	13–234	LEHHHHHH	^41^
JN2118HU	***T. evansi* type B**	AJ870487	Variant surface glycoprotein	M	16–1428	LEHHHHHH	^42^

^a^The antigen GM6 was produced by 3 tandem repeats of the selected region.

The antigens included in this study consisted of the fragment of the above-listed proteins produced by heterologous expression in *Escherichia coli* (Table 1). A nickel-NTA column was used for the antigen protein purification step due to the poly-HisTag added at the antigen’s C-ter. The purity value for each of the antigens was above 90%.

### Evaluation of the coupling efficacy of the antigens of interest

**Figure 1:**
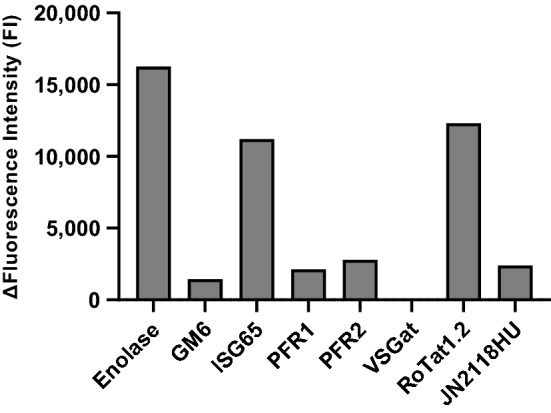
Evaluation of the coupling efficacy of the eight tested antigens to the fluorescent microspheres using anti-Histidine tag antibodies. The ΔFluorescence Intensity (FI) was determined as Fluorescence Intensity_Sample_—Fluorescence Intensity_Background_.

After optimisation of the experimental conditions, 5 µg of antigens were coupled to the magnetic microspheres with 1.25 × 10^6^ magnetic microspheres for enolase, PFR1, PFR2, VSGat, RoTat1.2 and JN2118HU. The amount of antigen GM6 coupled to 1.25 × 10^6^ magnetic microspheres was reduced to 0.5 µg. The efficacy of the coupling step was assessed using anti-polyhistidine tag antibodies. The ΔFluorescence Intensity (FI) values correspond to the difference in trimmed mean fluorescence intensity (MFI) between coupled microspheres incubated with 4 µg of anti-polyhistidine tag antibodies and coupled microspheres incubated with dilution buffer (Fig. 1). High ΔFluorescence Intensities were observed for enolase (16,258 FI), ISG65 (11,214 FI) and RoTat1.2. (12,307 FI). Antigens GM6, PFR1, PFR2 and JN2118HU displayed lower ΔFluorescence Intensities, from 1,449 FI to 2,795 FI. The ΔFluorescence Intensity observed for VSGat was the lowest, with 22 FI. This may be the result of either an issue during the coupling step that prevented the antigen from binding to the fluorescent microspheres, or a change in conformation of the antigen that limited access to the HisTag. The data provided by this analysis did not allow a conclusion on these hypotheses, so all eight antigens were retained for further experiments.

### Assessment of antigen reactivity

**Figure 2:**
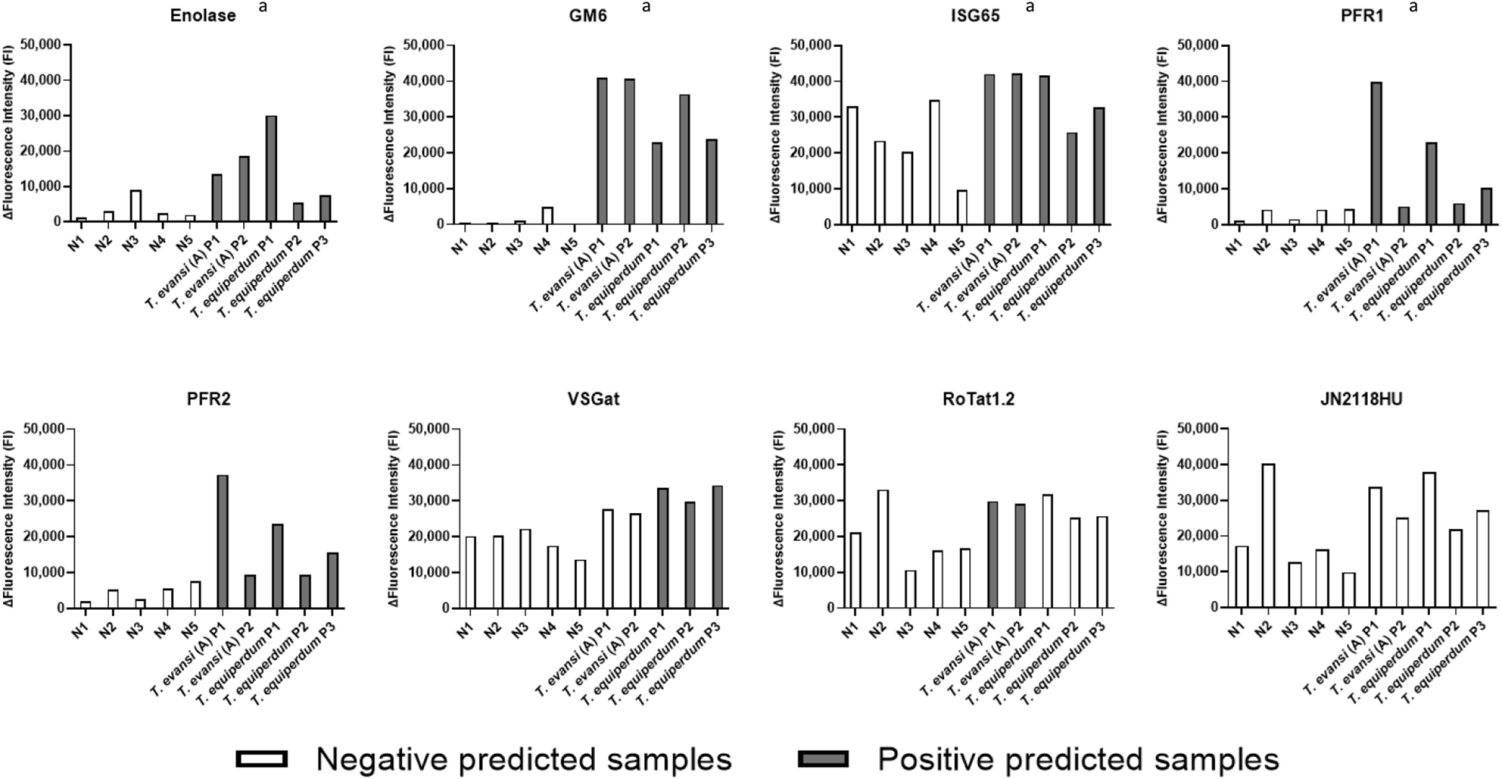
Serum reactivity of the eight antigens tested with ten reference sera. To assess the reactivity of the antigens, the coupled microspheres were incubated with ten different sera. Sera were selected for their immunological status: N = *Trypanozoon*-negative sample; *T. evansi* (A) Px = positive sample for *T. evansi* type A infection; *T. equiperdum* Px = positive sample for *T. equiperdum* OVI infection. ^a^ The antigen reactivity was assessed after the improvement of experimental conditions.

Ten sera were used to assess the diagnostic potential of the antigens: five negative sera (N1 to N5), two *T. evansi* type A positive samples (*T. evansi* (A) P1 and P2) and three *T. equiperdum* OVI positive sera (*T. equiperdum* P1, P2, and P3) (Fig. 2). The results obtained showed in particular high ΔFluorescence Intensities for negative samples using ISG65, VSGat, RoTat1.2 and JN2118HU, and low ΔFluorescence Intensities for positive samples using enolase, PFR1 and PFR2. In parallel, GM6 clearly discriminated between negative and positive samples. In keeping with these results, only GM6 was selected for the additional sensitivity and specificity tests. To optimise experimental conditions, different amounts of GM6 (0.1, 0.25, 0.5, 1, 2, 5, 25 and 50 µg) were tested during the coating step. The results obtained led us to use 0.5 µg of GM6 for further experiments.

### ROC, sensitivity and specificity analysis

**Figure 3:**
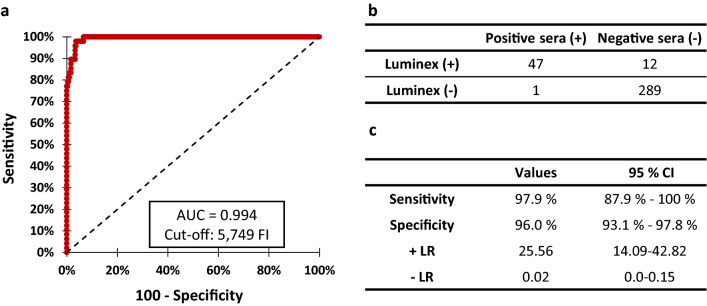
Sensitivity and specificity analysis for the GM6 antigen tested with a microsphere‐based immunoassay. (**a**) ROC analysis of the GM6 antigen using 301 negative sera and 48 positive sera. Cut-off value was determined at the MFI value giving the highest sensitivity plus specificity. (**b**) Contingency analysis of the 349 sera analysed in a microsphere‐based immunoassay. (**c**) Sensitivity, specificity values and Likelihood ratio (LR) determined for the microsphere‐based immunoassay using the GM6 antigen.

After optimising the experimental conditions as described in the material and methods, the experimental cut-off, sensitivity and specificity were determined using a receiver operating characteristics (ROC) analysis (Fig. 3). The sample set included 301 negative samples and 48 positive samples described in the Material and Methods section. The experimental cut-off of the microsphere‐based immunoassay determined by ROC analysis (corresponding to the mean fluorescence intensity (MFI) value giving the highest sensitivity and specificity in the ROC analysis) was 5,749 FI for antigen GM6 (Fig. 3a). The area under the curve (AUC) was determined to be 0.994. Of the 48 positive sera, 47 were identified as positive with the microsphere-based immunoassay and one was misidentified as a false negative sample (Fig. 3b). Of the 301 negative sera, 289 were negative with the microsphere-based immunoassay and 12 were false positives. Assays performed on panels of GM6 positive and negative sera showed a sensivity of 97.9% (95% CI: 87.9–100%) with a high specificity of 96.0% (95% CI: 93.1–97.8%) (Fig. 3c).

### Evaluation of GM6 on experimental sera

**Figure 4:**
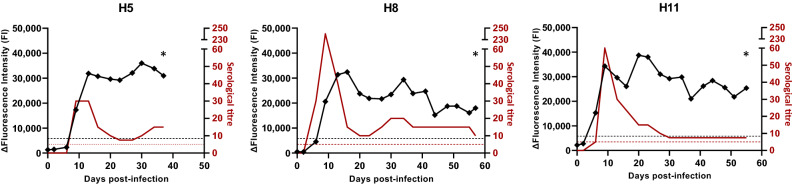
Detection of anti-GM6 antibodies from experimentally infected horse sera. Three Welsh pony mares, H5, H8 and H11, were infected with *T. equiperdum* OVI and monitored up to 57 days post infection by blood sampling at day 0 and then every 2–4 days. Serological titres (red line) were determined previously. ΔFluorescence Intensities were obtained with GM6-coupled microspheres in a microsphere‐based immunoassay and are shown by the black line. Dotted lines represent positive cut-offs for serological titres (red) and the GM6 assay (black). An asterisk indicates the end point of the experimental infection protocol.

Sera sampled from pony mares experimentally infected with *T. equiperdum* were analysed with a microsphere‐based immunoassay and compared with serological titres obtained by complement fixation test (CFT) (Fig. 4). The infection time courses ranged from 37 to 57 days post infection (dpi) depending on the experimental design. The inoculation of 5.0 × 10^4^ trypanosomes resulted in the development of infection characterised by dourine CFT seroconversions between 6 and 9 dpi. The serological titres determined by dourine CFT showed an immune response that reached 240 for H8 during the first fifteen days and that stabilised around 10 until the end point of the experiment. We observed detection of the anti-GM6 antibodies for the three pony mares between 6 and 9 days post infection. The first positive serological detections using CFT and a microsphere‐based immunoassay were concomitant for H5 and H11 on days 6 and 9 post infection respectively. Anti-GM6 antibodies were detected on 9 dpi for H8 whereas the CFT detected a positive result on 6 dpi. Moreover, a positive signal above 15,000 FI was detected by the microsphere‐based immunoassay throughout the experimental infection.

## Discussion

Recent epizootic infections by *Trypanozoon* in Europe demonstrate the real risk of importing equine trypanosomosis into non-endemic countries^10,11,19^. The development of sensitive and specific diagnostic tools is therefore essential to ensure the safety of the international horse trade. The transient presence of *Trypanozoon* parasites in the blood of equids complicates the direct detection of the parasites and explains why the OIE recommends the use of serological methods. This is the context which drove us to evaluate the ability of a microsphere‐based immunoassay using xMAP® technology to to test for equine trypanosomosis by assessing the diagnostic potential of eight eight different candidate antigens specific either to the *Trypanozoon* subgenus or to one specific clade.

The eight selected antigens were first coupled to different fluorescent microspheres in order to verify the efficacy of the coupling step using anti-HisTag antibodies. Out of the eight antigens, enolase, ISG65 and RoTat1.2 generated high fluorescence intensities (above 10,000 FI) suggesting effective coupling of the antigens. GM6, PFR1, PFR2 and JN2118HU generated signals between 1,400 FI and 2,800 FI while VSGat produced a very low fluorescence intensity of 22 FI. The relatively low fluorescence intensity obtained for these last five antigens could be explained either by a weak coupling of the antigens to the fluorescent microspheres or by the conformation of the coated antigens that made the HisTag inaccessible to the anti-HisTag antibodies. Discrepancies between the coating measurement with anti-HisTag antibodies and the efficacy of coupling antigens in diagnostic tests have also been described by others^18^, so we decided to perform serum reactivity assays for the eight antigens. The results obtained confirmed that coupling controls using anti-HisTag antibodies cannot accurately predict the reactivity of the antigen during these experiments since high fluorescence intensities were obtained for antigens with low fluorescence intensities using anti-HisTag antibodies (Figs. 1 and 2).

The sera reactivity tests performed on the eight antigens resulted in the exclusion of antigens ISG65, VSGat, Rotat1.2 and JN2118HU from our assays because of the high fluorescent signals obtained with negative sera (lack of specificity). Antigens enolase, PFR1 and PFR2 were also excluded due to low fluorescence intensities for positive sera (lack of sensitivity). The exclusion of seven out of the eight antigens originally selected in this study was unexpected, as several of these antigens (such as Rotat1.2^20^ and ISG65^18^) have already been used by other teams developing diagnostic tools for trypanosomosis. The reasons for the lack of efficacy remain unidentified. However, the purity of the recombinant antigens were about 90% that may lead to cross-reactions, by increasing the purity of the antigens we could observe an higher specificity of detection, but this implies a potential decrease of the sensitivity of the microsphere-based immunoassay. In addition, the heterologous expression in *Escherichia coli* (a prokaryote lacking the post-translational glycosylation system usually found in eukaryotes) probably altered the spatial conformation of our antigens, making them less specific for the *Trypanozoon* of interest. An alternative production system for recombinant antigens using a related trypanosomatid organism such as *Leishmania tarentolae*^21,22^ could be promising, but its cost is prohibitive. Another recombinant antigen production system using the yeast *Pichia pastoris* that has been shown to be efficient in the production of trypanosomal antigens could provide a balance between the lack of post-translational modifications with the antigen production in *E. coli* and the cost of producing recombinant antigens in *L. tarentolae*^23,24^.

Following the reactivity tests, only GM6 was selected for the sensitivity and specificity tests. This antigen seems robust enough to be a reliable target for the development of various diagnostic tools, since it has already been used for the development of an ELISA for *Trypanozoon* infections, an immunochromatographic test, and a microsphere-based immunoassay for human African trypanosomiasis^18,25–28^. The ability of this antigen to react specifically to antibodies from animals infected by *Trypanozoon* parasites is confirmed by the good specificity and sensitivity results (97.9% and 96.0% respectively). Among the 12 sera that gave false positive results, five sera from endemic areas of Northern Argentina were positive for equine infectious anemia and West Nile virus which could potentially result in cross-reactivity. The other seven sera were collected in France, where there is little probability of *Trypanozoon* infection in equids or of cross-reactivity as the country has been free of dourine for more than 50 years, suggesting that these sera are true negative. For these sera, we cannot exclude the hypothesis that their identification by xMAP® as positive samples results from cross-reactions with other *Trypanosoma* species such as *T. cruzi* or *T. vivax*^29^. We also observed that this test was able to detect *Trypanozoon* infections during their early stages, since it detected positive signals either concomitantly with or slightly after the immune response was detected by the CFT method.

One of the limitations of this microsphere‐based immunoassay for diagnosing *Trypanozoon* infections in equids is that the method is based on a single antigen specific to *Trypanozoon* parasites, since we had to exclude the three antigens selected for their theoretical clade specificity (VSGat, RoTat1.2 and JN2118HU). However, the multiplexing capability of this technique can be used in the future thanks to its evolutionary feature, which allows the addition of new antigen-coated microspheres to the current assay. Thus, if other antigens able to clearly detect all the *Trypanozoon* parasites or one species in particular are identified in the near future, they could actually be added to the assay as it stands today. As the antigen GM6 is shared by all *Trypanozoon* taxa, the immunoassay based on GM6-coated microspheres may eventually become applicable not only on equine but also on cattle, camels for example by varying the host specificity of the secondary antibody. In addition, several equine diseases are monitored through the reference laboratories’ network for international horse trade, which requires negative diagnostic results to allow the movement of horses. The technique’s multiplexing capability could be used to extend the diagnostic scope of this test to other equine diseases by combining, for example, the different tests already developed^30–32^ within a single multiplex test.

In conclusion, we have developed a specific and sensitive immunoassay based on GM6-coated microspheres for the serological diagnosis of *Trypanozoon* infections in equids. Although further investigations are still required to confirm the efficacy of the method on sera from naturally-infected horses, the detection of the anti-GM6 antibodies appears very promising and complementary to official serological methods prescribed by the OIE for the monitoring and control of *Trypanozoon* infections in equids. Its mutiplexing capability also suggests the future possibility of combining antigens specific to different equine pathogens to develop a single diagnostic tool for a broad and rapid diagnosis of a wide range of equine diseases.

## Material and methods

### Negative sera

Three hundred and one sera negative for equine trypanosomosis were included in this study. Of this total, 164 sera were collected between January 2015 and December 2017 in France (a region of France where dourine, surra and nagana were not recorded over the last 40 years), 99 were seropositive for equine piroplasmosis, 5 seropositive for equine viral arteritis, 3 seropositive for equine infectious anaemia and 5 seropositive for equine herpesvirus. Seven sera were from a sampling campaign performed in Argentina^33^ and 18 from a study involving the experimental infection of pony mares^34,35^.

### Positive sera

Forty-eight sera positive for equine trypanosomosis were included in this study. Of this total, 36 sera were from three endemic countries: Argentina (n = 34), Italy (n = 1) and Mongolia (n = 1). Their seropositivity was confirmed by dourine CFT or by RoTat1.2 trypanolysis. Twelve positive sera were sampled from 12 pony mares during an experimental infection protocol. Their seropositivity was confirmed by dourine CFT^34,35^.

### Experimental infections

As previously described, 12 Welsh pony mares (*Equus caballus*) initially negative for *Trypanozoon* infection, were infected with 5 × 10^4^ parasites of the *T. equiperdum* Onderstepoort Veterinary Institute (OVI) strain^34,35^. Serum samples were collected on day 0 and every 2 to 4 dpi. Three of the pony mares named H5, H8, and H11, were used for the study of the fluorescence intensity kinetics (n = 49). We randomly selected the pre-infection serum and only one post-infection serum from each experimentally infected pony mares for the sensitivity and specificity analysis.

All the experiments were conducted in accordance with the guidelines of directive 2010/63/EU of the European Parliament and of the Council, in the facilities of the Plateforme d’Infectiologie Expérimentale: PFIE, UE-1277, INRA Centre Val de Loire, Nouzilly, France. All the experimental procedures were ethically approved by the Loire Valley ethical review board (DGRI agreement APAFIS#2,015,010,908,456,425).

### Complement fixation test

Complement fixation tests (CFTs) were performed according to the prescribed OIE protocol^36^. Serological titres were determined according to the CFT analysis using the percentage of haemolysis inhibition and the dilution factor of positive wells.

### Coupling antigens with magnetic microspheres

The selected antigens were produced by heterologous expression in *Escherichia coli* and purified using a nickel-NTA column by Genecust® (Luxembourg). The antigens thus produced were coupled to Magplex® microspheres using the “Antibody Coupling Kit” (Luminex Corporation, Austin, TX, USA) according to the manufacturer’s instructions. Briefly, 1.25 × 10^6^ magnetic microspheres (BioPlex Pro Magnetic COOH Beads, Bio-Rad Laboratories) were washed three times with 500 µL of activation buffer. To activate the microspheres, 10 µL of the N-hydroxysulforsuccinimide sodium salt solution (S-NHS, 50 mg/mL, supplied in the kit) and 10 µL of 1-ethyl-3-(3-dimethylaminopropyl) carbodiimide hydrochloride (EDC, 50 mg/mL, Sigma-Aldrich) was added to 480 µL of activation buffer. The solution was incubated for 20 min on a rotary shaker at 40 rpm in the dark. After three washes with 500 µL of activation buffer, 0.1–50 µg of antigens were incubated with the microspheres using a total volume of 500 µL of solution for 2 h at 40 rpm. After optimisation, 1.25 × 10^6^ magnetic microspheres were coupled to 5 µg of enolase, PFR1, PFR2, VSGat, RoTat1.2 or JN2118HU. For GM6, 0.5 µg of antigens were coupled to 1.25 × 10^6^ magnetic microspheres. The coupled microspheres were washed three times with wash buffer before resuspension in 1 mL of wash buffer. The microsphere concentrations were determined by counting under a microscope and stored at 4 °C in the dark until use.

### Luminex assay

Serum samples were diluted at 1:200 in dilution buffer (PBS-BSA 2%) before heat inactivation for 30 min at 56 °C ± 2 °C. After washing the 96-well plate with the dilution buffer, 2,500 coupled magnetic microspheres were placed in each well (Bio-Plex ProTM flat bottom well plates, Bio-Rad Laboratories). Fifty µL of serum samples or dilution buffer (negative control) were added to the microspheres for 1 h at room temperature (RT) on a plate shaker at 800 rpm in the dark. The plate was washed three times with a washing solution containing PBS-Tween 0.05% using a magnetic plate. 50 µL of secondary biotinylated goat anti-horse IgG (Jackson Immuno Research Inc.) diluted at 1:500 in the dilution buffer was added to each well for 45 min at room temperature (RT) with shaking at 800 rpm in the dark. After three washing steps, 50 µL of streptavidin R-phycoerythrin conjugate (SAPE; 1 µg/mL; Bio-Rad Laboratories) was added to each well at a dilution of 1:100 in the dilution buffer for 15 min at RT on a plate shaker. After three washes, the mixture was resuspended in 100 µL of drive fluid solution (Luminex Corporation, Austin, TX, USA) and the fluorescence intensities of at least 50 microspheres were monitored on the MagPix system using xPONENT software. To reduce the fluorescence intensity background, the experimental conditions were optimised by replacing the dilution buffer by a commercial solution (Blocking solution, Candor) and the plate was pre-washed with the dilution buffer.

### Data analysis

A receiver operating characteristic (ROC) analysis was carried out using XLSTAT software (Addinsoft). Figures were generated using GraphPadPrism9.2 software.

### Ethics statement

The authors confirm that the ethical policies of the journal, as noted on the journal’s author guidelines page, have been adhered to. All experiments were conducted in accordance with the guidelines of directive 2010/63/EU of the European Parliament and Council, in the facilities of the Plateforme d’Infectiologie Expérimentale: PFIE, UE-1277, INRA Centre Val de Loire, Nouzilly, France. All experimental procedures were ethically approved by the Loire Valley ethical review board (DGRI agreement APAFIS#2,015,010,908,456,425).

## Data Availability

All the data generated or analysed during this study are included in this published article. The raw data sets used during the present study are kept at the ANSES Animal Health Laboratory (Normandy site) and are available upon reasonable request.

## References

[1] FAOSTAT. http://www.fao.org/faostat/en/#data/QA/visualize, 2021).

[2] Pritchard JC (2010). Animal traction and transport in the 21st century: Getting the priorities right. Vet. J..

[3] Burn CC, Dennison TL, Whay HR (2010). Environmental and demographic risk factors for poor welfare in working horses, donkeys and mules in developing countries. Vet. J..

[4] OIE. Chapter 7.12. *Welfare of working equids*. World organisation for Animal Health. Organisation Mondiale de la Santé Animale, Paris, pp. 1–10 (2017).

[5] Stringer A (2014). Improving animal health for poverty alleviation and sustainable livelihoods. Vet. Rec..

[6] Büscher P (2019). Equine trypanosomosis: Enigmas and diagnostic challenges. Parasit Vectors.

[7] Barrett MP (2003). The trypanosomiases. Lancet.

[8] Brun R, Hecker H, Lun ZR (1998). Trypanosoma evansi and *T. equiperdum*: Distribution, biology, treatment and phylogenetic relationship (a review). Vet. Parasitol..

[9] Radwanska M, Vereecke N, Deleeuw V, Pinto J, Magez S (2018). Salivarian trypanosomosis: A review of parasites involved, their global distribution and their interaction with the innate and adaptive mammalian host immune system. Front. Immunol..

[10] Gutierrez C, Desquesnes M, Touratier L, Büscher P (2010). *Trypanosoma evansi*: Recent outbreaks in Europe. Vet. Parasitol..

[11] Pascucci I (2013). Diagnosis of dourine in outbreaks in Italy. Vet. Parasitol..

[12] Carnes J (2015). Genome and phylogenetic analyses of Trypanosoma evansi reveal extensive similarity to *T. brucei* and multiple independent origins for dyskinetoplasty. PLoS Negl. Trop. Dis..

[13] Oldrieve G, Verney M, Jaron KS, Hébert L, Matthews KR (2021). Monomorphic *Trypanozoon*: Towards reconciling phylogeny and pathologies. Microb. Genom..

[14] Claes F (2005). Comparison of serological tests for equine trypanosomosis in naturally infected horses from Kazakhstan. Vet. Parasitol..

[15] Zablotskij VT (2003). The current challenges of dourine: Difficulties in differentiating *Trypanosoma equiperdum* within the subgenus *Trypanozoon*. Rev. Sci. Tech..

[16] Kellar KL (2001). Multiplexed fluorescent bead-based immunoassays for quantitation of human cytokines in serum and culture supernatants. Cytometry.

[17] Vignali DA (2000). Multiplexed particle-based flow cytometric assays. J. Immunol. Methods.

[18] Nzou SM (2016). Development of multiplex serological assay for the detection of human African trypanosomiasis. Parasitol. Int..

[19] Desquesnes M (2008). First outbreak of *Trypanosoma evansi* in camels in metropolitan France. Vet. Rec..

[20] Tehseen S (2015). Parasitological, serological and molecular survey of *Trypanosoma evansi* infection in dromedary camels from Cholistan Desert, Pakistan. Parasit Vectors.

[21] Ferrer MJ (1955). Production of recombinant *Trypanosoma cruzi* antigens in *Leishmania tarentolae*. Methods Mol. Biol..

[22] Rooney B, Piening T, Büscher P, Rogé S, Smales CM (2015). Expression of *Trypanosoma brucei* gambiense antigens in *Leishmania tarentolae*. Potential for use in rapid serodiagnostic tests (RDTs). PLoS Negl. Trop. Dis..

[23] Birhanu H (2015). Surra Sero K-SeT, a new immunochromatographic test for serodiagnosis of *Trypanosoma evansi* infection in domestic animals. Vet. Parasitol..

[24] Rogé S (2013). Recombinant expression of trypanosome surface glycoproteins in *Pichia pastoris* for the diagnosis of *Trypanosoma evansi* infection. Vet. Parasitol..

[25] Boulangé A (2017). Development of a rapid antibody test for point-of-care diagnosis of animal African trypanosomosis. Vet. Parasitol..

[26] Davaasuren B (2017). The evaluation of GM6-based ELISA and ICT as diagnostic methods on a Mongolian farm with an outbreak of non-tsetse transmitted horse trypanosomosis. Vet. Parasitol..

[27] Mizushima D (2018). The utility of an rTeGM6-4r-based immunochromatographic test for the serological diagnosis of non-tsetse-transmitted equine trypanosomosis in rural areas of Mongolia. Parasitol Res..

[28] Thuy NT, Goto Y, Lun ZR, Kawazu S, Inoue N (2012). Tandem repeat protein as potential diagnostic antigen for *Trypanosoma evansi* infection. Parasitol Res..

[29] Ramírez-Iglesias JR, Eleizalde MC, Reyna-Bello A, Mendoza M (2017). Molecular diagnosis of cattle trypanosomes in Venezuela: Evidences of *Trypanosoma evansi* and *Trypanosoma vivax* infections. J. Parasit Dis..

[30] Balasuriya UB (2006). Detection of antibodies to West Nile virus in equine sera using microsphere immunoassay. J. Vet. Diagn. Invest..

[31] Beck C (2015). A high-performance multiplex immunoassay for serodiagnosis of Flavivirus-associated neurological diseases in horses. Biomed. Res. Int..

[32] Laroucau K (2020). Development of a microsphere-based immunoassay for the serological detection of glanders in equids. Acta Trop..

[33] Hébert L (2021). Serological evidence of equine infectious anaemia, West Nile fever, surra and equine piroplasmosis in a herd of horses in northern Argentina. Vet. Parasitol. Reg. Stud. Rep..

[34] Hébert L (2018). Validation of a new experimental model for assessing drug efficacy against infection with *Trypanosoma equiperdum* in horses. Vet. Parasitol..

[35] Hébert L (2018). Melarsomine hydrochloride (Cymelarsan®) fails to cure horses with *Trypanosoma equiperdum* OVI parasites in their cerebrospinal fluid. Vet. Parasitol..

[36] OIE. *Terrestrial animal health code*. World organisation for Animal Health. Organisation Mondiale de la Santé Animale, Paris (2021).

[37] Li Z (2020). An unbiased immunization strategy results in the identification of enolase as a potential marker for nanobody-based detection of *Trypanosoma evansi*. Vaccines (Basel).

[38] Berriman M (2005). The genome of the African trypanosome *Trypanosoma brucei*. Science.

[39] Abdille MH, Li SY, Ding J, Suo X (2008). *Trypanosoma evansi*: Paraflagellar rod protein 1 and 2 are similar but lack common B cell epitopes. Exp. Parasitol..

[40] Luciani M (2018). *Trypanosoma equiperdum* low molecular weight proteins as candidates for specific serological diagnosis of dourine. Front. Vet. Sci..

[41] Verloo D (2000). Comparison of serological tests for *Trypanosoma evansi* natural infections in water buffaloes from north Vietnam. Vet. Parasitol..

[42] Cuypers B (2017). Genome-wide SNP analysis reveals distinct origins of *Trypanosoma evansi* and *Trypanosoma equiperdum*. Genome Biol. Evol..

